# Stroke care in people with and without schizophrenia: a retrospective, observational study

**DOI:** 10.1192/j.eurpsy.2022.740

**Published:** 2022-09-01

**Authors:** J. Quarenta, M. Gonçalves-Pinho, A. Freitas, S. Nascimento Ferreira

**Affiliations:** 1 Centro Hospitalar do Tâmega e Sousa, Departamento De Psiquiatria E Saúde Mental, Penafiel, Portugal; 2 CINTESIS - Center for Health Technology and Services, Faculty of Medicine, University of Porto, Department Of Community Medicine, Information And Health Decisions Sciences, Porto, Portugal

**Keywords:** stroke, schizophrénia, comorbidity

## Abstract

**Introduction:**

Serious mental illness tends to course with a higher prevalence of comorbidities and schizophrenia is a disabling disease that affects approximately 1% of the world population. Worldwide, cerebrovascular accidents are an important cause of mortality and disability and in Portugal they are one of the leading causes of death in the general population. There is growing evidence that the prevalence of strokes is higher in people with schizophrenia, with pronounced age and gender variations.

**Objectives:**

To describe the sociodemographic and clinical differences among patients hospitalized with a primary diagnosis of cerebrovascular disease with and without a secondary diagnosis of schizophrenia in Portugal.

**Methods:**

We performed a retrospective observational study using a natiowide hopitalization database containing all hosptalizations registered in Portuguese hosptals from 2008 to 2015. Based on the International Classification of diseases version 9, clinical modification, hospitalizations with a primary diagnosis of stroke were selected (431;433;434), and from those, the ones with a secundary diagnosis of schizophrenia (295.xx) were isolated for a sociodemographic and clinical comparative study. Comorbidities were analysed using the Chalson index score.

**Results:**

Episodes associated with a secondary diagnosis of schizophrenia were younger (mean: 66 vs 73.7 years; p<0.001) and had longer median LoS (10.0 vs 8.0 days; p<0.001). In-hospital mortality was lower in patients with schizophrenia (11.7% vs 13.2%).

**Conclusions:**

The understanding of the association of cerebrovascular accidents with schizophrenia is complex. Although some studies show conflicting evidence, more attention should be given to the investigation of the incidence, prevalence and impact of cerebrovascular diseases within this particular population.

**Disclosure:**

No significant relationships.

